# Integrated multi-omics analysis identifies PPAR signaling network associations with metabolic-immune dysregulation and intestinal injury in PDCoV-infected piglets

**DOI:** 10.1080/21505594.2026.2739013

**Published:** 2026-09-27

**Authors:** Xingchen Guo, Chao A, Yijie Ding, Yudan Zhang, Haijuan Zhang, Jinyong Wang, Yiyu Zhang, Tao Wang

**Affiliations:** a College of Animal Science/Key Laboratory of Animal Genetics, Breeding and Reproduction in the Plateau Mountainous Region, Ministry of Education, Guizhou University, Guiyang, China; b Department of Pig Production, Chongqing Academy of Animal Science, Rongchang, Chong Qing, China; c Department of Pig Production, National Center of Technology Innovation for Pigs, Rongchang, Chong Qing, China

**Keywords:** Porcine deltacoronavirus (PDCoV), multi-omics integration, PPAR signaling pathway, interferon response, metabolic reprogramming, host-pathogen interaction

## Abstract

Porcine deltacoronavirus (PDCoV) is an emerging enteric coronavirus that causes severe diarrhea and high mortality in neonatal piglets and has demonstrated the capacity to infect human cells. However, the host response networks that govern disease outcome remain poorly understood. Here, we performed integrated transcriptomic, proteomic, and metabolomic profiling of jejunal tissues from PDCoV-infected piglets, representing the first such analysis in the primary target organ of this pathogen. Multi-omics integration identified a core host-response network characterized by concurrent activation of a potent type I interferon-mediated antiviral response and notable alterations in lipid metabolism. The peroxisome proliferator-activated receptor (PPAR) signaling pathway was among the most significantly enriched pathways, marked by coordinated upregulation of *LPL*, *FABP3*, and *SCD* and downregulation of *SLC27A2*. Metabolomic analysis demonstrated widespread degradation of membrane glycerophospholipids and alterations in the arachidonic acid–prostaglandin inflammatory axis, as evidenced by substrate consumption and accumulation of the pro-inflammatory mediator 20-hydroxy-PGE2. We propose a hypothetical working model in which PDCoV infection is associated with PPAR network modulation, potentially contributing to lipid resource reallocation and the establishment of a pro-inflammatory microenvironment that may exacerbate intestinal injury. Notably, similarities can be observed between these PDCoV-associated alterations and the metabolic-immune dysregulation reported in severe acute respiratory syndrome coronavirus 2 (SARS-CoV-2) infection, raising the hypothesis of shared pathogenic features across coronaviruses. Our findings highlight the PPAR pathway and its downstream lipid mediators as potential therapeutic targets warranting further investigation, and suggest that existing PPAR agonists may merit evaluation as candidate drugs for repurposing in future studies.

## Introduction

Porcine deltacoronavirus (PDCoV), an emerging enteropathogenic coronavirus, has posed a significant threat to the global swine industry since its discovery [1–7]. This pathogen causes acute diarrhea, vomiting, and dehydration in neonatal piglets, leading to high mortality rates and substantial economic losses [8]. The jejunum, a primary site of PDCoV infection and replication, is particularly susceptible [9]. Notably, compared to the more extensively studied porcine epidemic diarrhea virus (PEDV), PDCoV exhibits a broader cellular tropism, including the demonstrated capacity to infect human cells in vitro [10–16]. This characteristic raises considerable public health concerns regarding its zoonotic potential. Despite its economic and biological importance, the molecular mechanisms underlying PDCoV pathogenesis remain incompletely elucidated. A critical knowledge gap exists in understanding how the virus disrupts intestinal barrier function and evades host immune responses, which hinders the development of effective countermeasures.

Current understanding of PDCoV-host interactions largely relies on transcriptomic, proteomic, or metabolomic analyses conducted in isolation, with few studies integrating these layers to reveal the coordinated host response [17–21]. For instance, while several studies have documented the differential expression of host genes involved in innate immunity and inflammation following PDCoV infection [17,18], and others have identified viral proteins that interact with host factors such as nucleoproteins [19], these single-omics approaches provide a fragmented perspective. Moreover, most of these analyses have been performed in cell lines, which cannot fully recapitulate the complex tissue microenvironment and multicellular interactions that shape the host response in vivo. Critically, they fail to reveal how viral infection systematically reprograms host metabolic networks, a key aspect of pathogenesis, as metabolic reprogramming enables viruses to acquire biosynthetic resources and drive tissue damage [22]. Consequently, these approaches cannot capture the complex, multi-layered regulatory cascade that connects initial viral recognition to the final pathological outcome. A systems-level understanding, which integrates changes across genetic regulation, protein function, and metabolic activity within the relevant tissue context, is urgently needed to bridge this gap.

To address this, we employed a physiologically relevant neonatal piglet model, overcoming the limitations of in vitro systems in mimicking the intestinal microenvironment. We hypothesized that PDCoV orchestrates a pathogenic program through coordinated disruption of the jejunal transcriptome, proteome, and metabolome, culminating in a dysregulated “immune-metabolic-barrier” network. To test this, we conducted an integrated multi-omics analysis of infected jejunum using RNA sequencing (RNA-seq), data-independent acquisition (DIA)-based quantitative proteomics, and liquid chromatography-mass spectrometry (LC-MS)-based untargeted metabolomics. This work delivers the first comprehensive multi-omics landscape of the host response in the primary target organ of PDCoV, systematically delineating its mechanistic basis. Our findings not only provide an unprecedented panoramic view of PDCoV-induced pathology but also pinpoint key host dependency factors and metabolic vulnerabilities as potential targets for novel therapeutic strategies.

## Results

### PDCoV infection model validation and jejunal pathological changes

The successful establishment of the PDCoV infection model was confirmed through a comprehensive assessment encompassing characteristic clinical signs, gross lesions, robust viral replication, and associated histopathological and ultrastructural damage in the jejunum. Following viral challenge, all infected piglets developed typical signs of viral enteritis within 48 hours, with symptoms progressively worsening until the experimental endpoint at 72 hours post-infection (hpi). The clinical presentation was characterized by a 100% incidence of profuse watery diarrhea and lethargy, accompanied by intermittent vomiting in 75% of the animals. Consistent with these clinical signs, postmortem examination revealed diffuse intestinal pathology. The jejunal walls were notably thin and translucent, with evident effacement of mucosal folds. Serosal petechial hemorrhages were observed, and the intestinal lumen was filled with copious yellowish, watery fluid (Figure 1(A)).

**Figure 1.**
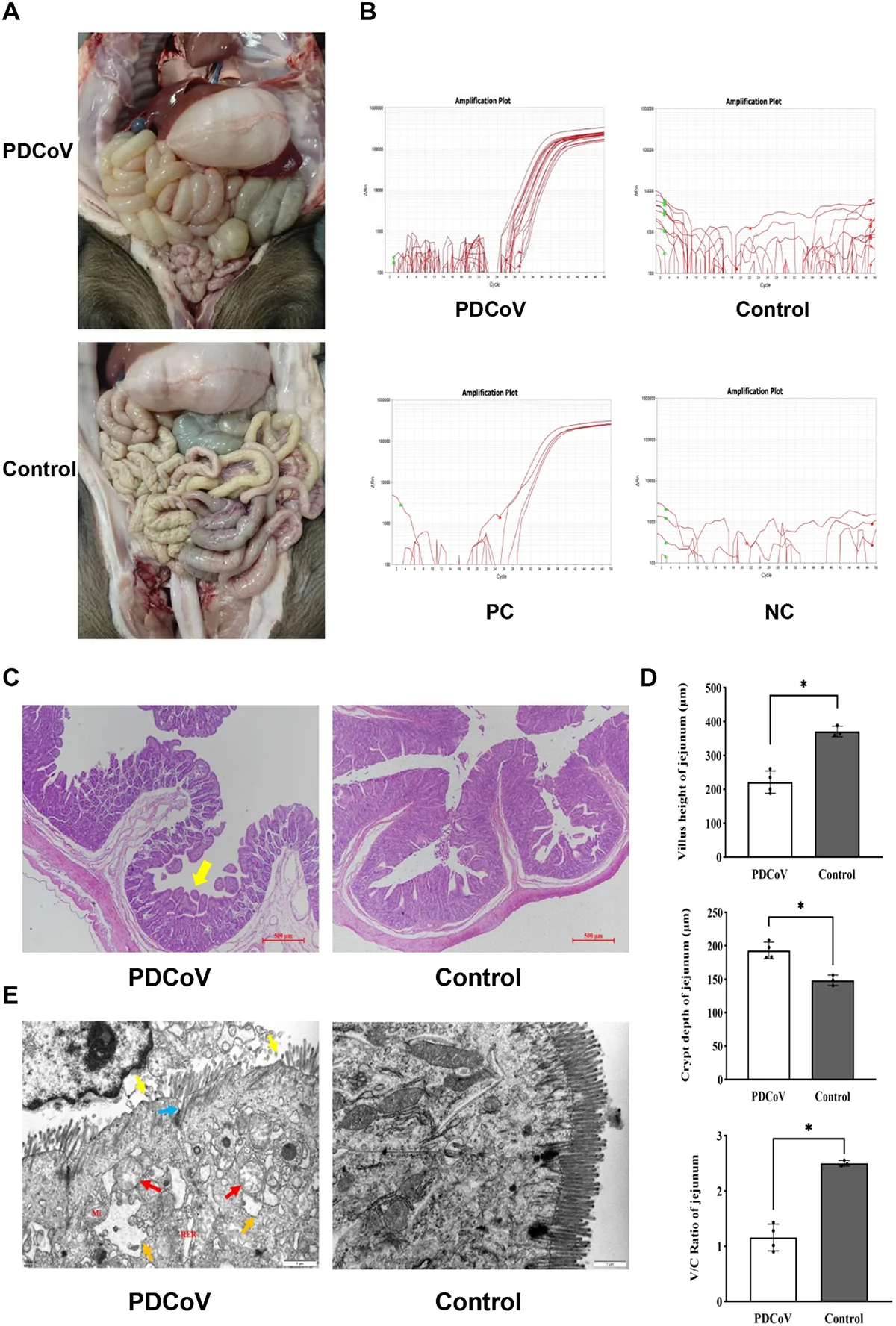
PDCoV infection causes severe jejunal damage in piglets. (A) the autopsy symptoms of jejunum in control and infected piglets. (B) detection of PDCoV in fecal samples by probe‑based quantitative real-time PCR (qPCR). Positive control (pc), negative control (NC). (C) representative hematoxylin and eosin (H&E) staining of jejunal sections. Yellow arrows indicate villous shedding and fragmentation. Scale bar = 500 µm. (D) quantitative morphometric analysis of villus height, crypt depth, and villus height-to-crypt depth ratio (V/C) in jejunal sections from control and PDCoV-infected piglets. Data are presented as mean ± sd. *n* = 3 for control group, *n* = 4 for PDCoV-infected group. *p < 0.05 (Student’s *t*-test). (E) representative transmission electron microscopy (TEM) images of intestinal epithelial cells. Mi, mitochondria; RER, rough endoplasmic reticulum. Red arrows indicate markedly swollen mitochondria; yellow arrows indicate disrupted cristae and vacuolization; orange arrows indicate conspicuously dilated RER; blue arrow indicates focal disintegration of tight junctions.

To molecularly confirm the infection, we performed qualitative PCR (qPCR) with a PDCoV-specific probe on fecal samples. This analysis demonstrated substantial viral shedding in four of the five challenged piglets, while no viral signal was detected in the control group (Figure 1(B)), thereby verifying a productive PDCoV infection. The remaining piglet (No. 1) showed low and poorly reproducible viral shedding in fecal samples and was therefore excluded from subsequent multi-omics analysis (see Sample collection and selection for multi-omics analysis). Histopathological examination of H&E-stained jejunal sections further corroborated the severe damage. Compared to the well-preserved, finger-like villi in controls, the infected jejunum exhibited extensive villous shedding and fragmentation (Figure 1(C)). Morphometric analysis further confirmed these histopathological observations. Quantitative measurements revealed that villus height was significantly reduced in PDCoV-infected piglets compared with controls, while crypt depth was markedly increased, resulting in a significantly decreased villus height-to-crypt depth ratio (Figure 1(D)). To elucidate the subcellular basis of the pathology, we conducted transmission electron microscopy (TEM). Ultrastructural analysis uncovered severe injury to the intestinal epithelial cells, characterized by markedly swollen mitochondria with disrupted cristae and vacuolization, as well as conspicuously dilated rough endoplasmic reticulum. The epithelial barrier integrity was compromised, evidenced by focal disintegration of tight junctions. Furthermore, the brush border was profoundly disorganized, with microvilli appearing shortened, sparse, and irregularly arranged (Figure 1(E)).

Taken together, these comprehensive clinical, pathological, and virological test results support the successful establishment of the PDCoV infection model and demonstrate that PDCoV infection is associated with severe multi-level structural damage to the porcine jejunum.

### Transcriptomic signatures of the PDCoV-infected host jejunum

To characterize host jejunal gene expression in response to PDCoV infection, we performed RNA-seq analysis on jejunal tissues from PDCoV-infected and control piglets. RNA sequencing generated approximately 85.1 to 96.2 million raw reads per sample. After quality filtering, each library yielded 12.4 to 14.0 Gb of clean data, with Q30 scores ranging from 95.37% to 95.89% (Table S1). The clean reads were then mapped to the swine reference genome (Sus_scrofa. Sscrofa11.1), with alignment rates between 94.98% and 95.54% (Table S2). The high depth, quality scores, and mapping rates indicate that the resulting transcriptomic data are suitable for downstream differential expression analysis.

Principal component analysis (PCA) revealed a clear separation between the PDCoV-infected and control groups, indicating substantial transcriptomic differences induced by infection (Figure 2(A)). Comparative analysis identified 544 differentially expressed genes (DEGs), with 249 upregulated and 295 downregulated in the infected group. The distribution of these DEGs is displayed in a volcano plot, which highlights genes based on their fold change and statistical significance (Figure 2(B)). Furthermore, hierarchical clustering of the DEGs showed distinct expression patterns that consistently distinguished the infected samples from the controls (Figure 2(C)).

**Figure 2.**
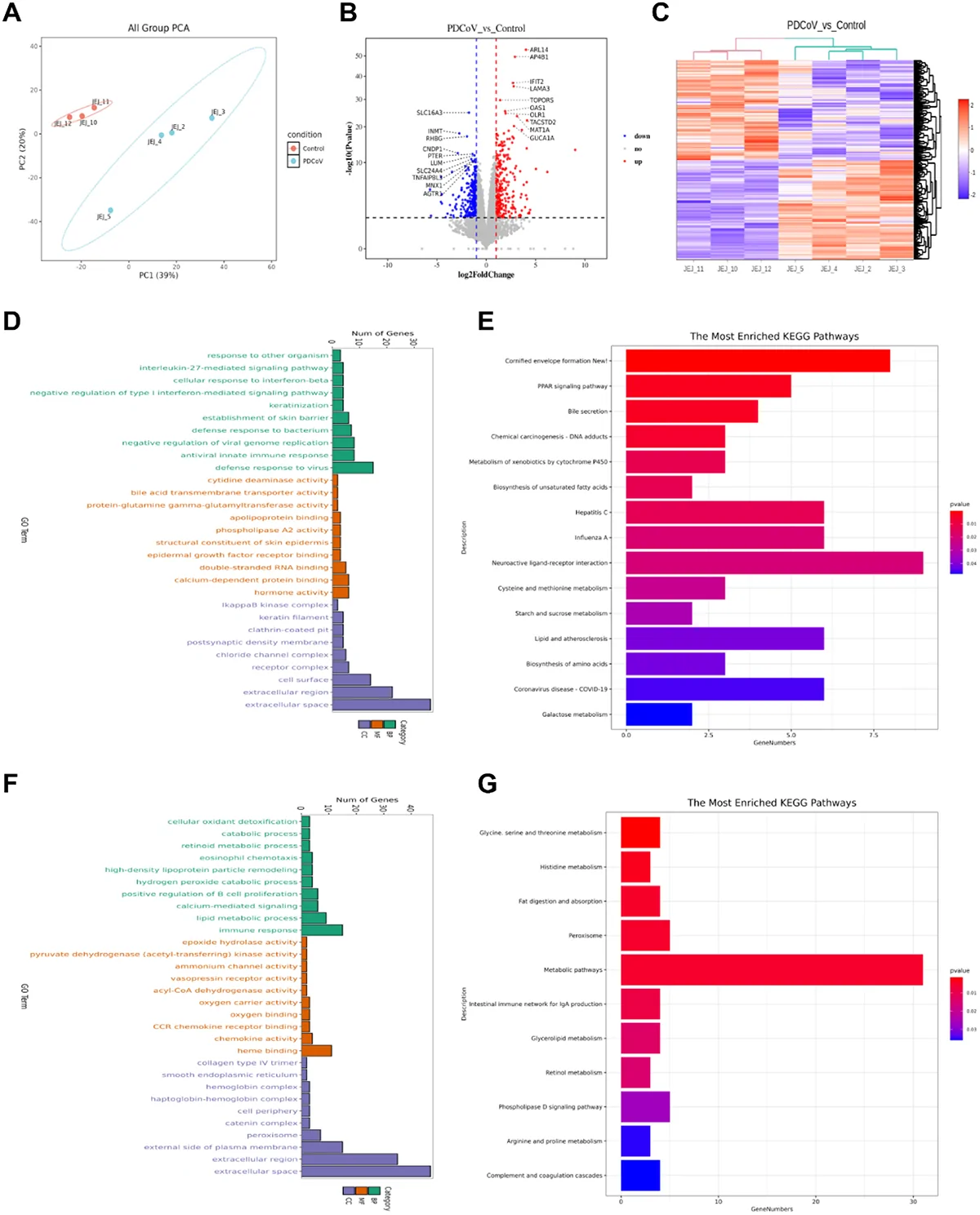
Transcriptomic analysis of jejunal tissues from PDCoV-infected and control piglets. (A) principal component analysis (PCA) showing separation between control and PDCoV‑infected groups. (B) volcano plot of differentially expressed genes (DEGs). Red and blue dots represent significantly upregulated and downregulated genes, respectively (|log_2_FC| >1, *p* < 0.05, DESeq2 with Benjamini-Hochberg correction). (C) hierarchical clustering analysis of DEGs. (D, E) GO and KEGG enrichment analyses of up‑regulated DEGs. (F, G) GO and KEGG enrichment analyses of down‑regulated DEGs. *n* = 3 for control group, *n* = 4 for PDCoV-infected group.

Gene Ontology (GO) and Kyoto Encyclopedia of Genes and Genomes (KEGG) pathway enrichment analyses were performed on the up-regulated and down-regulated DEGs separately. These analyses revealed a distinct pattern characterized by concurrent immune activation and metabolic suppression.

The up-regulated DEGs were significantly enriched in immune and barrier functions. GO terms such as defense response to virus and antiviral innate immune response were prominent (Figure 2(D)). Consistent with this, KEGG analysis highlighted enrichment in immune-related pathways including Coronavirus disease-COVID-19 and Influenza A. Additionally, pathways related to barrier integrity (Cornified envelope formation) and metabolic regulation (PPAR signaling pathway, Bile secretion) were also enriched (Figure 2(E)).

Conversely, the down-regulated DEGs were overwhelmingly associated with metabolic processes. KEGG analysis showed significant suppression of pathways for Fat digestion and absorption and Glycerolipid metabolism (Figure 2(G)). GO analysis corroborated this finding, revealing downregulation in terms like lipid metabolic process, oxygen carrier activity, and acyl-CoA dehydrogenase activity (Figure 2(F)).

Collectively, the transcriptomic profile indicates that PDCoV infection is associated with a robust activation of antiviral and barrier defense pathways, coupled with a broad suppression of core metabolic functions in the host jejunum.

### DIA-based proteomic profiling of the PDCoV-infected host jejunum

DIA-proteomics was performed on jejunal tissues from PDCoV-infected and control pigs. Across samples, we identified 8,689–8,915 proteins (Fig. S1). PCA confirmed clear separation between the two groups (Figure 3(A)). Comparative analysis identified 646 differentially expressed proteins (DEPs), with 324 upregulated and 322 downregulated. The distribution and expression patterns of these DEPs are visualized in a volcano plot (Figure 3(B)) and a hierarchical clustering heatmap (Figure 3(C)).

**Figure 3.**
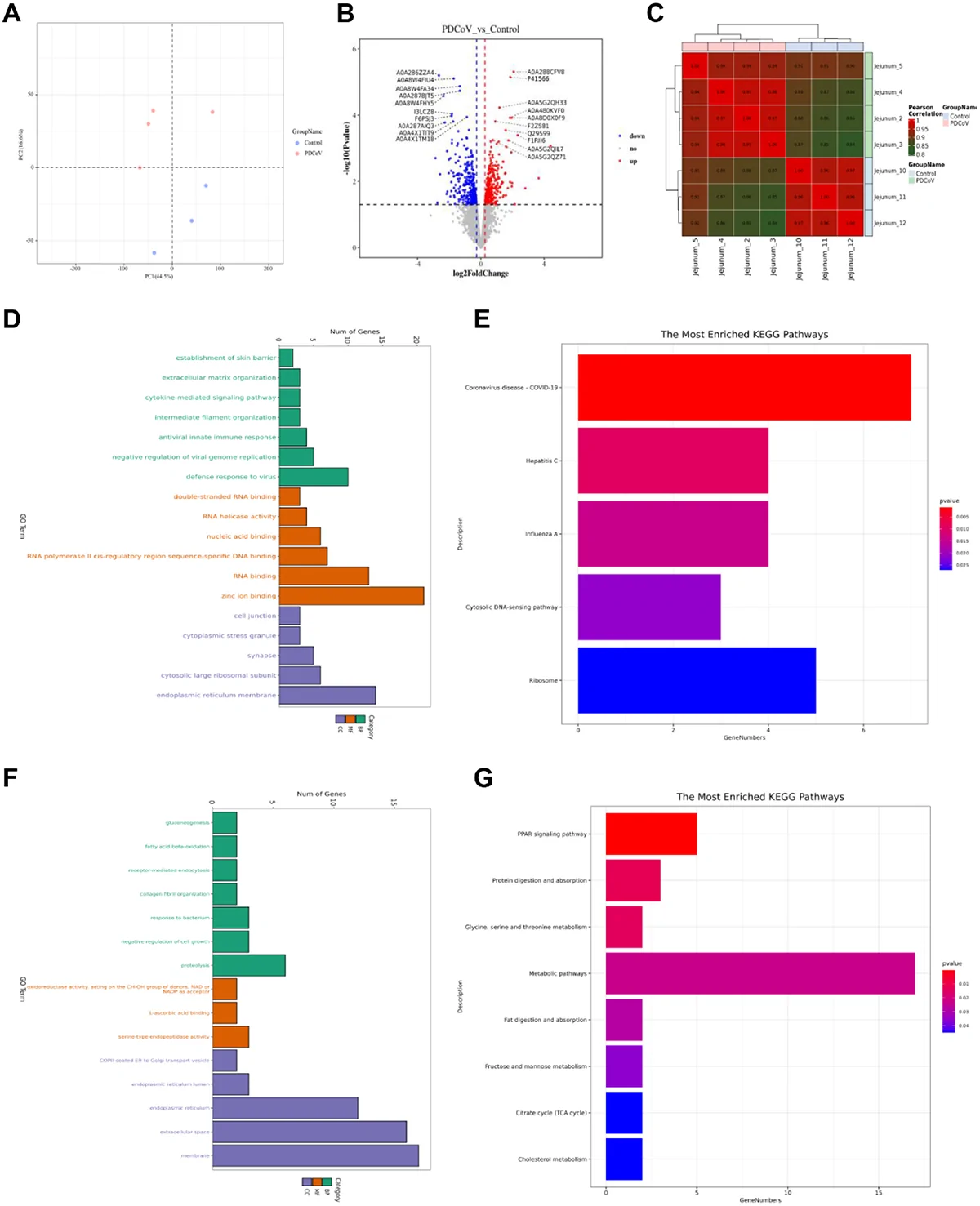
DIA-based proteomic analysis of jejunal tissues from PDCoV-infected and control piglets. (A) principal component analysis (PCA) showing separation between control and PDCoV‑infected groups. (B) volcano plot of differentially expressed proteins (DEPs). Red and blue dots represent significantly upregulated and downregulated proteins, respectively (|log_2_FC| >0.263, *p* < 0.05, limma package). (C) hierarchical clustering analysis of DEPs. (D, E) GO and KEGG enrichment analyses of up‑regulated DEPs. (F, G) GO and KEGG enrichment analyses of down‑regulated DEPs. *n* = 3 for control group, *n* = 4 for PDCoV-infected group.

To functionally contextualize the DEPs, we performed enrichment analysis. Upregulated DEPs were significantly enriched in immune-related processes. GO analysis highlighted terms including antiviral innate immune response, defense response to virus, and cytokine-mediated signaling pathway. Barrier-related functions such as establishment of skin barrier and extracellular matrix organization were also enriched among upregulated proteins, aligning with transcriptomic data (Figure 3(D)). KEGG pathway analysis further indicated activation of Coronavirus disease – COVID-19, Influenza A, and the Cytosolic DNA-sensing pathway (Figure 3(E)).

In contrast, downregulated DEPs were predominantly associated with metabolic suppression. KEGG analysis revealed broad downregulation of metabolic pathways, including specific suppression of PPAR signaling pathway, Fat digestion and absorption, Glycine, serine and threonine metabolism, and the Citrate cycle (TCA cycle) (Figure 3(G)). GO analysis confirmed the downregulation of key metabolic processes such as fatty acid beta-oxidation and gluconeogenesis, suggesting a systemic disruption of energy and lipid metabolism (Figure 3(F)).

### Untargeted metabolomic analysis of the PDCoV-infected host jejunum

Untargeted metabolomics was performed on jejunal tissues from PDCoV-infected and control pigs in both positive (ESI^+^) and negative (ESI^−^) ionization modes. The total ion chromatograms (TICs) displayed distinct metabolic profiles between groups (Figure 4(A, B)). PCA revealed a clear separation of the two groups (Figure 4(C)). An Orthogonal Partial Least Squares Discriminant Analysis (OPLS-DA) model further confirmed this separation (Figure 4(D)). The model was validated by 200 permutation tests (*R*^2^*Y* = 0.999, *Q* [2] = 0.817, *p* < 0.005), indicating robustness and low risk of overfitting (Figure 4(E)). Comparative analysis identified 225 differentially expressed metabolites (DEMs), with 58 upregulated and 167 downregulated (Figure 4(F)). Unsupervised hierarchical clustering of these DEMs showed distinct expression patterns that effectively distinguished infected from control samples (Figure 4(G)).

**Figure 4.**
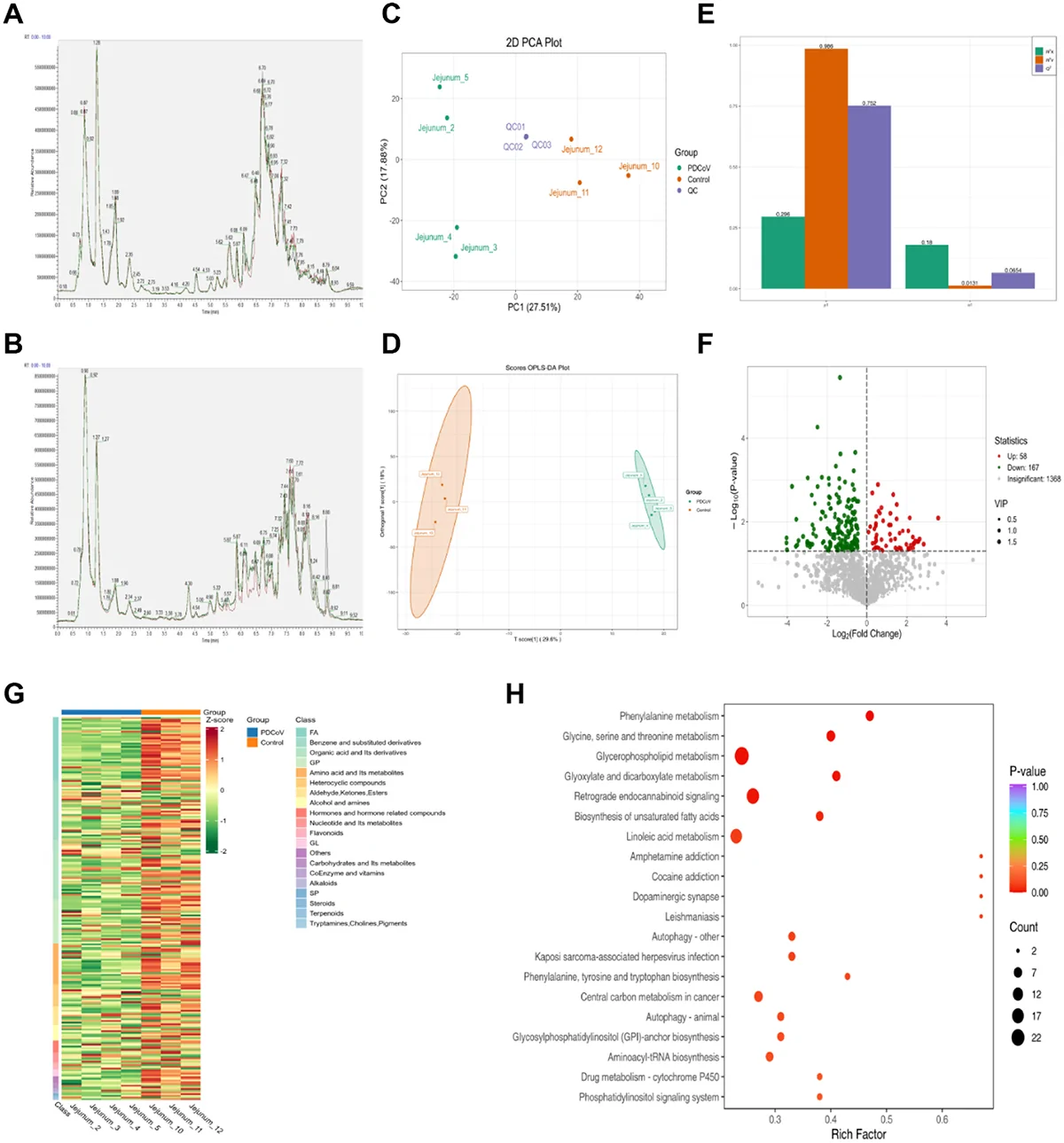
Untargeted metabolomic analysis of jejunal tissues from PDCoV-infected and control piglets. (A, B) total ion chromatograms (TICs) in positive ESI^+^ and negative ESI^−^ electrospray ionization modes. (C) principal component analysis (PCA) showing separation of metabolic profiles between control and PDCoV-infected groups. (D) Orthogonal partial least squares discriminant analysis (OPLS‑DA) of metabolic profiles. (E) validation of the OPLS‑DA model by 200 permutation tests. (F) volcano plot of differentially expressed metabolites (DEMs). Red and blue dots represent significantly upregulated and downregulated metabolites, respectively (VIP > 1, *p* < 0.05). (G) hierarchical clustering analysis of DEMs. (H) KEGG enrichment analyses of DEMs. *n* = 3 for control group, *n* = 4 for PDCoV-infected group.

KEGG pathway enrichment analysis of the DEMs highlighted significant perturbations in several core metabolic pathways. These included glycerophospholipid metabolism, biosynthesis of unsaturated fatty acids, glycine, serine and threonine metabolism, and phenylalanine metabolism (Figure 4(H)). These metabolomic findings identified the specific metabolic pathways that were altered during PDCoV infection.

### Integrated transcriptomic and proteomic analysis reveals a core host-response network

Integrative analysis of the transcriptome and proteome identified a core network of 79 molecules consistently dysregulated during PDCoV infection, representing high-confidence effectors of the host response (Figure 5(A, B)). The overall correlation between mRNA and protein abundance was significant (*R* = 0.47, Figure 5(C)). A nine-quadrant analysis detailed this relationship: the majority of molecules showed concordant expression (72 in Q3 and 75 in Q7), while a subset exhibited discordant (23 in Q1/Q9) or mRNA-only changes (75 in Q4/Q6), suggesting additional post-transcriptional regulation (Figure 5(D)).

**Figure 5.**
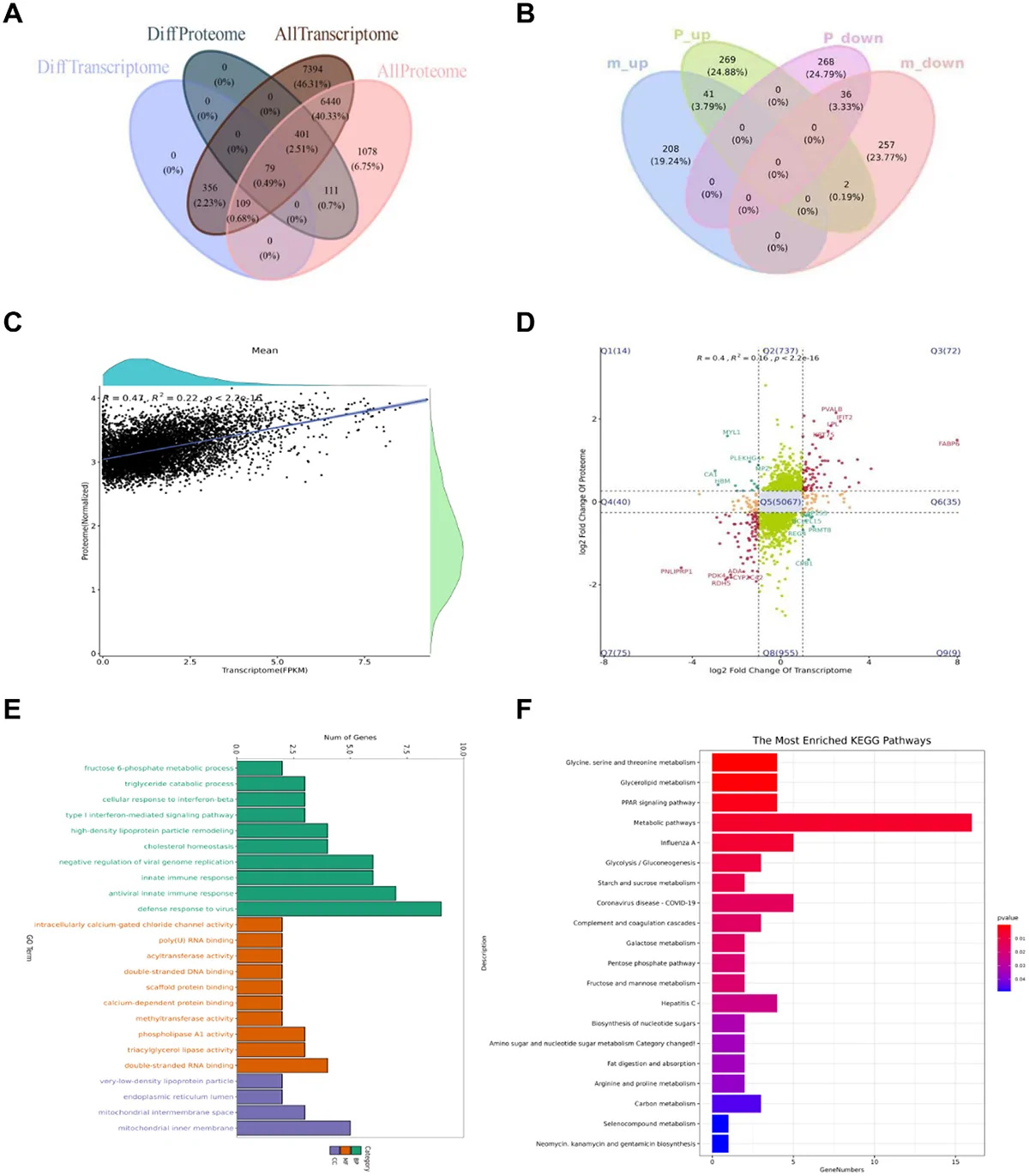
Integrative transcriptome and proteome analysis of PDCoV‑infected and control piglets. (A, B) core dysregulated molecule network identified by integrative analysis of transcriptomic and proteomic data (79 molecules consistently dysregulated at both mRNA and protein levels). (C) Pearson correlation between mRNA and protein abundance. (D) nine‑quadrant analysis of mRNA and protein expression. (E, F) GO and KEGG enrichment analyses of the 79 core dysregulated molecules. *n* = 3 for control group, *n* = 4 for PDCoV-infected group.

GO and KEGG pathway enrichment analysis of the core 79 molecules revealed their simultaneous involvement in two major biological programs: a robust antiviral immune response and a substantial reprogramming of host metabolism (Figure 5(E, F)). Among these, the antiviral immune response was mainly driven by *MX1*, *MX2*, *OAS1*, *OAS2*, *OASL*, *RSAD2*, *IFIT2*, *IFIT5*, *ISG15*, and *DHX58*; the PPAR signaling pathway was driven by *LPL*, *FABP3*, *SCD*, *SLC27A2*, *PLTP*, *OLR1*, *ACADL*, and *PPARG*. Additionally, glycine, serine and threonine metabolism was enriched in the core network, driven by *GAMT*, *GATM*, *PHGDH*, *GCAT*, and *ALAS2*. This integrated analysis thus identifies a core host-response network concurrently engaged in antiviral defense and metabolic reprogramming, providing a basis for further functional investigation.

### The antiviral interferon response is robustly activated in the core network

A focused examination of the immune component within the 79-molecule core network confirmed the potent activation of the innate antiviral system. Key effector molecules of the interferon (IFN) response, including classical interferon-stimulated genes (ISGs) such as *MX1*, *MX2*, *OAS1*, *OAS2*, *RSAD2*, and *IFIT2*, were uniformly and significantly upregulated in PDCoV-infected jejunum (Figure 6(A)). This coherent upregulation pattern was further validated by the significant enrichment of pathways including type I interferon-mediated signaling pathway and defense response to virus within this subset. The consistent induction of these canonical ISGs within the high-confidence core network demonstrates the establishment of a potent, cell-intrinsic antiviral state in the intestinal epithelium upon PDCoV infection.

**Figure 6.**
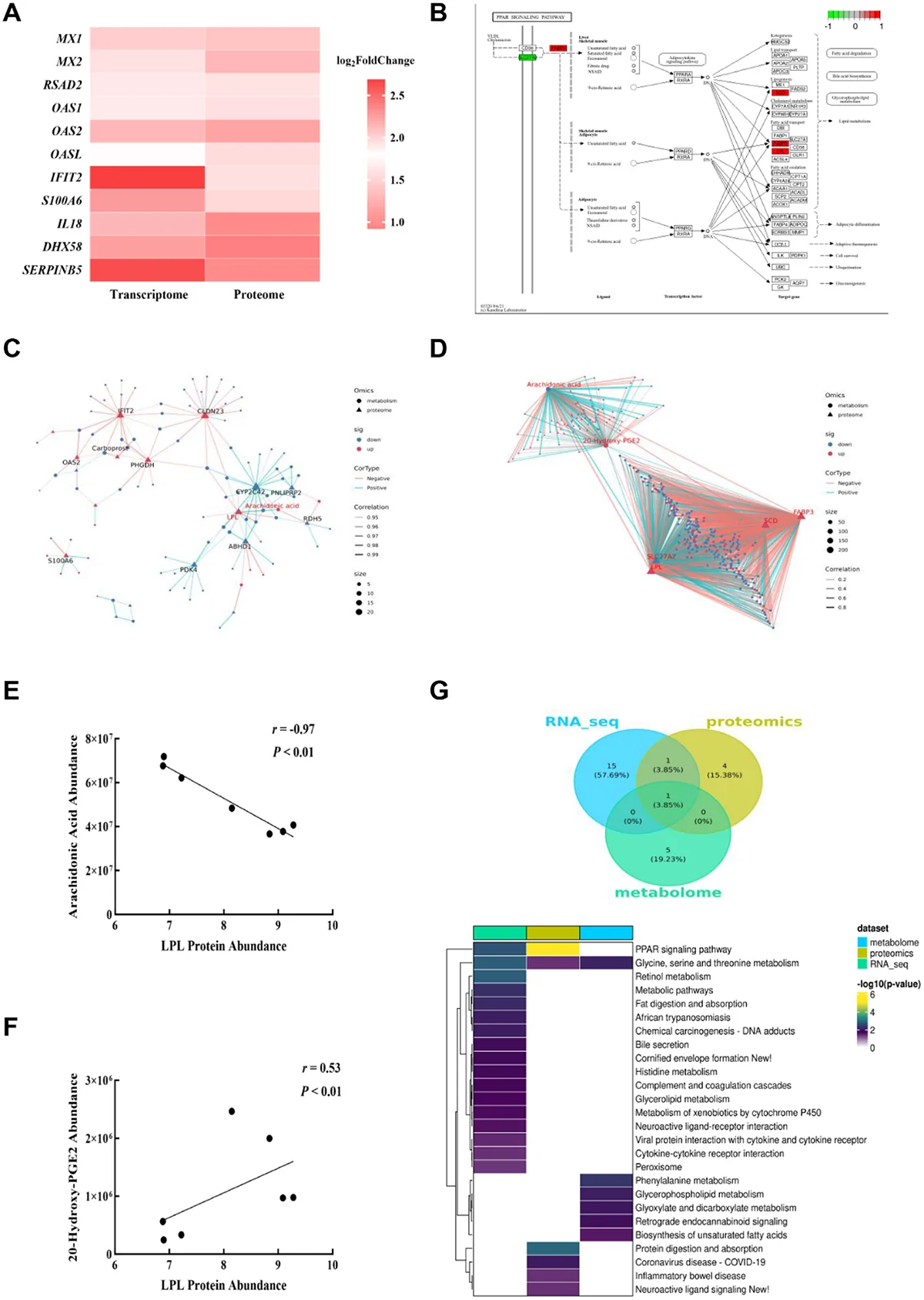
Analysis of immune response, metabolic pathways and multi-omics correlation in PDCoV-infected and control piglets. (A) expression levels of key interferon-stimulated genes (ISGs) in the core molecule network. (B) perturbation of key constituents in the PPAR signaling pathway. (C) global correlation network of multi-omics layers (|*r*| >0.95, *p* < 0.05). (D) focused subnetwork of the PPAR signaling hub and its linked metabolites (|*r*| >0.2, *p* < 0.05). (E, F) quantitative correlation analysis of LPL protein with arachidonic acid and 20-hydroxy-PGE2 (Pearson correlation). (G) cross-omics KEGG pathway enrichment analysis showing pathways significantly altered in at least two omics layers. *n* = 3 for control group, *n* = 4 for PDCoV-infected group.

### The PPAR signaling pathway as a key pathway associated with metabolic reprogramming

Conversely, the metabolic arm of the core network was dominated by the significant enrichment of the PPAR signaling pathway. A detailed dissection of this pathway within our dataset revealed a highly coordinated perturbation pattern among its key constituents (Figure 6(B)). Specifically, genes/proteins involved in extracellular lipid hydrolysis and processing were upregulated, including LPL (lipoprotein lipase), FABP3 (fatty acid-binding protein 3), and SCD (stearoyl-CoA desaturase). In contrast, SLC27A2 (solute carrier family 27 member 2), which facilitates long-chain fatty acid import, was downregulated. This precise, bidirectional modulation of enzymes involved in lipid uptake, transport, and modification suggests a specific and coordinated modulation of the host lipid metabolic machinery.

### Multi-omics integration supports a functional metabolic-immune axis

To systematically explore functional connections between the transcriptional/translational regulation and the metabolic phenotype, we performed a pairwise correlation analysis between the 79 core molecules (integrated from transcriptome and proteome) and all DEMs.

A global correlation network revealed extensive and statistically significant interactions between the multi-omics layers (Figure 6(C); |*r*| >0.95, *p* < 0.05). This unbiased analysis revealed broad crosstalk, with metabolites connecting to both immune-related molecules (e.g. *IFIT2*, *OAS2*) and metabolic enzymes. Notably, this network highlighted that arachidonic acid, a central inflammatory precursor, served as a key hub interacting with numerous proteins.

Focusing on the core story, we extracted the PPAR signaling hub and its linked metabolites to construct a focused subnetwork (Figure 6(D)). This visualization clearly delineated the coordinated perturbation within the lipid metabolic axis: upregulated effectors of lipid processing (*LPL*, *FABP3*, *SCD*) were centrally connected to arachidonic acid and its downstream product 20-hydroxy-PGE2, while the downregulated fatty acid transporter *SLC27A2* was peripherally located.

Within this tightly correlated subnetwork, the PPAR pathway component LPL emerged as a critical node bridging the metabolic shift to a pro-inflammatory outcome. Quantitative correlation analysis revealed that LPL protein abundance exhibited a strong negative correlation with its putative substrate, arachidonic acid (*r* = −0.97, *p* < 0.01), and a strong positive correlation with the pro-inflammatory metabolite 20-hydroxy-PGE2 (*r* = 0.53, *p* < 0.01) (Figure 6(E, F)). This positions LPL within a reprogrammed metabolic network, where its upregulation is linked to substrate depletion (AA) and is associated with the accumulation of a pro-inflammatory mediator.

Finally, cross-omics KEGG pathway enrichment analysis provided systems-level support. Cross-omics integration identified the PPAR pathway and glycine, serine and threonine metabolism as the only pathways enriched in at least two omics layers (Figure 6(G)). This analysis confirmed a coherent vertical axis: the PPAR signaling pathway, perturbed at the gene/protein level, was functionally linked to downstream glycerophospholipid metabolism and biosynthesis of unsaturated fatty acids in the metabolome. This integrated evidence supports the PPAR-associated metabolic alterations as a central feature of the host response to PDCoV infection.

Altogether, these analyses support the existence of a functional metabolic-immune axis, in which PPAR-associated metabolic alterations are quantitatively linked to both upstream transcriptional regulation and downstream inflammatory metabolite production, highlighting its potential importance in PDCoV pathogenesis.

### Experimental validation of key hub molecules

To independently validate the central molecular signatures identified in our multi-omics analysis, we performed quantitative verification at the tissue levels.

First, we confirmed the expression changes of key targets in the infected jejunum tissue. Quantitative PCR analysis validated the significant upregulation of interferon-stimulated genes (*IFIT2*, *MX1, OASL*, *RSAD2*) and the core metabolic genes *LPL*, *FABP3*, and *SCD* at the mRNA level, while *SLC27A2* was confirmed to be downregulated (Figure 7(A)). Western blot analysis further demonstrated a consistent increase in the protein abundance of MX1, RSAD2 and FABP3, in the PDCoV-infected group compared to controls (Figure 7(B)). Additionally, enzyme-linked immunosorbent assay (ELISA) was conducted to verify the levels of betaine, creatine, arachidonic acid, and prostaglandin E2 (PGE2) in jejunal tissues (Figure 7(C)).

**Figure 7.**
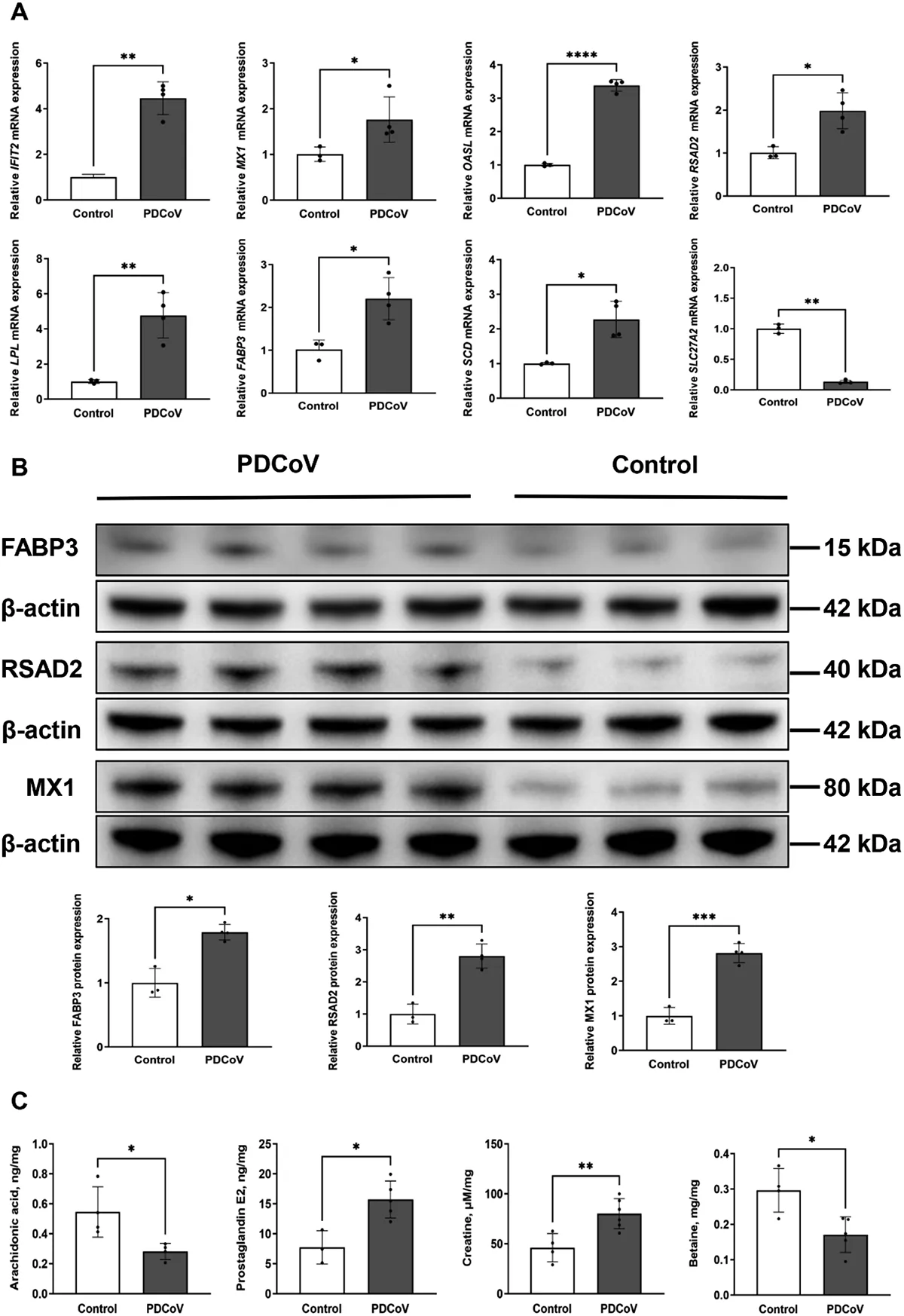
Experimental validation of key hub molecules in PDCoV‑infected and control piglets. (A) mRNA expression levels of key genes (*IFIT2, MX1*, *OASL, RSAD2*, *LPL, FABP3*, *SCD, SLC27A2*) detected by quantitative real-time PCR (qPCR). (B) protein expression levels of MX1, RSAD2 and FABP3 detected by Western blotting. β-actin was used as a loading control. (C) concentrations of arachidonic acid (AA), prostaglandin E2 (PGE2), creatine, and betaine in jejunal tissues detected by enzyme-linked immunosorbent assay (ELISA). For (A to C), values are presented as mean ± sd. *n* = 4 biological replicates for the infected group, *n* = 3 biological replicates for the control group. Multiple technical replicates were performed for each sample. Significant differences compared with the control group: * *p* < 0.05, **p < 0.01, ***p < 0.001, ****p < 0.0001, Student’s *t*-test.

Collectively, these validation experiments verify the in vivo expression patterns of the core network components, key metabolites, and signaling molecules, further supporting the reliability of our omics data.

### An integrated model of PDCoV-induced pathogenesis

Synthesizing the multi-omics evidence, we propose a hypothetical working model (Figure 8). In this model, PDCoV infection is associated with a dual-track host response: a robust, canonical interferon-mediated antiviral defense and notable alterations in the PPAR metabolic hub. The coordinated changes within the PPAR pathway correlate with a lipid metabolic landscape characterized by two key functional associations: (i) structural compromise through the widespread depletion of membrane phospholipids, and (ii) inflammatory exacerbation via a potential metabolic flux from arachidonic acid to pro-inflammatory prostaglandins. This cascade, along with the ongoing immune response, may contribute to the disruption of intestinal epithelial homeostasis, potentially underpinning the resultant diarrheal disease.

**Figure 8.**
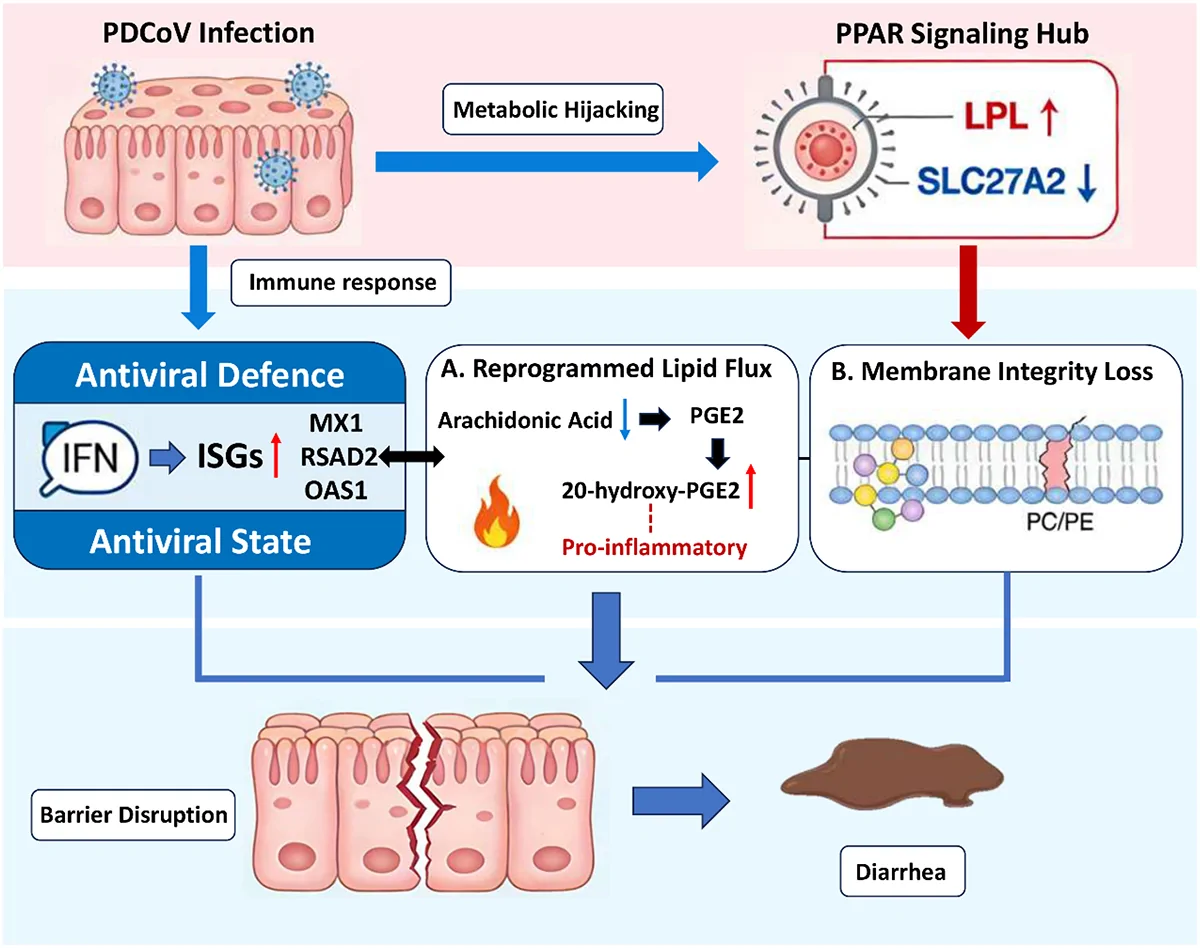
A hypothetical working model illustrating PPAR-associated metabolic-immune alterations during PDCoV infection. Based on integrative multi-omics evidence, PDCoV infection is associated with a dual-track host response: a robust type I interferon-mediated antiviral defense (left) and PPAR-associated lipid metabolic alterations (right). The coordinated changes in the PPAR pathway correlate with membrane phospholipid degradation and arachidonic acid (AA) release, potentially leading to increased production of pro-inflammatory prostaglandins (e.g. 20-hydroxy-PGE2). This model is hypothesis-generating and does not imply established causality.

## Discussion

This study provides the first integrated multi-omics dissection of the host response to PDCoV infection in the neonatal piglet jejunum. By converging transcriptomic, proteomic, and metabolomic data, we define a core host-response network that molecularly intertwines two major, concurrent events: a potent type I interferon-mediated antiviral response and a notable reprogramming of lipid metabolism, with the PPAR signaling pathway identified as a significantly enriched pathway. Functionally, this metabolic rewiring is linked to widespread membrane phospholipid degradation and alterations in the AA-prostaglandin inflammatory axis. Our data support a model in which PDCoV pathogenesis is associated with concurrent immune-metabolic dysregulation, a pattern similar to that reported for SARS-CoV-2 infection in humans [23,24]. This convergence raises the possibility of shared pathogenic features across coronaviruses, despite substantial differences in host species, tissue tropism, disease manifestations, and viral biology between PDCoV and SARS-CoV-2 [25,26]. The similarities observed should therefore be viewed as hypothesis-generating observations that warrant further investigation, rather than as evidence of evolutionarily conserved mechanisms.

As the initial defense mechanism of the host, the innate immune system initiates intracellular signaling cascades upon pattern recognition receptors (PRRs) recognition of pathogen-associated molecular patterns (PAMPs), leading to the transcriptional induction of pro-inflammatory cytokines, type I interferons, and antiviral effectors that coordinate pathogen clearance [27–29]. Our analysis demonstrates that PDCoV infection is associated with a robust intrinsic immune defense, with classical ISGs such as *MX1*, *OAS1*, and *RSAD2* markedly upregulated (Figure 6(A)). This aligns with multiple studies on PDCoV infection in both in vivo and in vitro [17–20,30]. Collectively, these convergent findings across different cell types (IPEC-J2, ST, HIEC-6, and our in vivo jejunum) support that activation of the type I interferon axis is a consistent response to PDCoV infection. Given the critical role of the IFN pathway in initiating antiviral resistance and shaping subsequent adaptive immunity, PDCoV appears to have evolved mechanisms to antagonize its induction and signaling to establish productive infection. The presence of this strong antiviral backdrop raises a critical question: how does PDCoV successfully replicate despite this hostile environment? Our integrated data suggest the answer lies in a parallel strategy targeting host metabolism.

Host cell metabolism is essential for viral replication by supplying necessary metabolites such as amino acids, fatty acids, and nucleotides. Many viruses are known to alter host metabolism to optimize their biosynthetic needs [22]. Although glycine, serine and threonine metabolism showed the broadest multi-omics coverage, we focused on the PPAR signaling pathway because of its direct functional relevance to lipid metabolism and immune regulation, processes that are central to PDCoV pathogenesis. In this context, a striking finding of our study is the significant enrichment of the PPAR signaling pathway associated with PDCoV infection metabolic reprogramming. PPARs belong to the nuclear receptor superfamily and function as transcription factors that regulate multiple metabolic pathways, including lipid and glucose metabolism, energy homeostasis, and cell differentiation [31,32]. Additionally, they play a vital role in innate immune responses [33–37]. Specifically, we observed coordinated upregulation of *LPL*, *FABP3*, and *SCD* concurrent with downregulation of *SLC27A2*, suggesting an alteration in lipid metabolism. This pattern suggests a shift away from direct fatty acid import and toward increased hydrolysis of circulating lipid complexes and enhanced de novo synthesis of monounsaturated fatty acids, metabolic adaptations that show similarities to those observed in SARS-CoV-2-induced lipid reprogramming in human lung [23,36,37]. For enveloped viruses, host lipids are indispensable for viral envelope formation, replication complex assembly, and spike protein palmitoylation, processes that are critical for viral entry and assembly [24,38–40]. SARS-CoV-2 has been shown to modulate PPARα activity to alter lipid metabolism, contributing to lipotoxicity and respiratory dysfunction [23,36,37]. Similarly, our data suggest that PDCoV infection is associated with modulation of the PPAR pathway, which may contribute to the accumulation of specific lipid precursors, raising the hypothesis that PPAR signaling may represent a common feature in coronavirus infection.

Extending these observations, Su et al. recently reported a “non-Warburg effect” in PDCoV-infected IPI-21 cells, characterized by pyruvic acid accumulation driven by enhanced pyruvate kinase activity and glutaminolysis to support pyrimidine synthesis [41]. Wang et al. further identified ferroptosis as a key pathway in PDCoV-infected IPEC-J2 cells, demonstrating that the ferroptosis activator erastin inhibits viral replication [17]. Building upon these findings, the PPAR-associated lipid alterations identified in our study may represent an upstream factor contributing to these metabolic changes. Pyruvic acid accumulation is likely attributable, at least in part, to enhanced fatty acid oxidation via PPARα activation, which provides acetyl-CoA for pyruvate synthesis while concurrently suppressing TCA cycle flux [36,42]. Furthermore, membrane phospholipid degradation and lipid peroxidation resulting from PPAR dysregulation may synergize with ferroptosis to exacerbate intestinal epithelial damage [37]. Critically, our in vivo data extend these in vitro observations by demonstrating that PDCoV-associated metabolic alterations operate within the complex intestinal tissue microenvironment, with the PPAR pathway emerging as a potentially central pathway. This suggests that tissue-level integration of epithelial and immune cell responses may contribute to this metabolic signature. The widespread membrane phospholipid degradation and AA-PGE2 axis activation observed in our study are consistent with a potential connection between metabolic reprogramming and inflammatory pathology, thereby complementing and extending previous in vitro findings.

The metabolomic data reveal striking parallels with COVID-19 pathophysiology, particularly regarding membrane disruption and inflammatory lipid mediator production. Glycerophospholipid downregulation in PDCoV-infected jejunum signifies cellular membrane disintegration (Figure 4(H)), a phenomenon analogous to SARS-CoV-2-induced endothelial disruption and alveolar-capillary barrier dysfunction [43–48]. This membrane degradation likely serves as the primary source of AA release. However, alternative explanations for AA release should also be considered. Beyond PPAR-associated metabolic alterations, generalized membrane degradation resulting from viral cytopathic effects or secondary inflammatory responses could independently contribute to AA liberation and subsequent prostaglandin synthesis. As a key bioactive lipid, AA is released from membrane phospholipids by phospholipase A2 during inflammatory responses [49–51] and serves as a precursor for various inflammatory mediators, including prostaglandins and leukotrienes [52,53]. While these mediators activate local immune defenses against pathogens, excessive AA metabolism can trigger uncontrolled inflammation, leading to tissue damage. This is evidenced by the concurrent depletion of AA and accumulation of its downstream metabolite 20-hydroxy-PGE2, revealing activation of a pro-inflammatory effector pathway also observed in COVID-19. Notably, sustained AA metabolic activation may further impair phospholipid synthesis, creating a vicious cycle that exacerbates membrane damage and intestinal barrier dysfunction. SARS-CoV-2 infection is similarly associated with activation of the AA cascade, leading to overproduction of prostaglandins and leukotrienes that contribute to cytokine storms and tissue injury [51,54,55]. PGE2, in particular, functions as a “cytokine amplifier” that can enhance viral replication and suppress host defenses [50,56,57]. The strong statistical correlation between LPL and these metabolic shifts collectively suggests that PPAR-associated lipid metabolism may play a contributing role in this pro-inflammatory axis, highlighting its potential as a candidate worthy of further investigation.

Our findings suggest a conceptual framework in which immune activation and metabolic alterations are linked in coronavirus pathogenesis, with parallels observed between PDCoV and SARS-CoV-2. PDCoV infection is associated with a strong interferon response and concurrent alterations in the PPAR-mediated metabolic network, which may support viral replication and contribute to pathological sequelae. This framework raises the hypothesis that infection may be associated with modulation of host metabolic resources, potentially contributing to the insufficiency of antiviral defenses. Consistent with this model, SARS-CoV-2 appears to employ a similar strategy in COVID-19, where PPAR manipulation supports replication and exacerbates inflammation through dysregulated lipid metabolism [23,36,37]. The resulting PGE2 overproduction amplifies immune cell recruitment and tissue damage, redirecting antiviral responses toward immunopathology [50,57]. Notably, PPARα knockout mice exhibit exacerbated pulmonary inflammation and impaired fatty acid oxidation, phenotypes that recapitulate severe pathology observed in both PDCoV-infected piglets and COVID-19 patients, thereby supporting the concept that PPAR signaling may play a role in coronavirus pathogenesis [58,59].

The conserved metabolic-immune dysregulation identified in this study may offer opportunities for further exploration for therapeutic intervention. Among the proposed targets, the PPAR signaling pathway represents a promising candidate, potentially linking lipid reprogramming, membrane degradation, and inflammation. PPAR agonists, including PPARα agonists (e.g. fenofibrate) and PPARγ agonists (e.g. pioglitazone), have shown promise in COVID-19 preclinical studies by reversing virus-induced metabolic changes, suppressing inflammation, and mitigating cytokine storms [24,60–63], although further investigation is warranted. Building on these insights, early targeting of PPAR in PDCoV infection could potentially normalize lipid metabolism and dampen the AA-prostaglandin axis, although this hypothesis requires direct experimental testing. For the AA cascade, inhibition of microsomal prostaglandin E synthase-1 (mPGES-1) represents a refined strategy that selectively blocks pro-inflammatory PGE2 synthesis while avoiding the adverse effects associated with global COX inhibition [51]. Given the shared features observed between PDCoV and COVID-19, future evaluation of these targets may be warranted as potential therapeutic strategies against PDCoV infection.

Several limitations of this study should be acknowledged. First, our multi-omics data are correlative and do not establish causality. Functional intervention studies, including PPAR agonist/antagonist treatment or gene knockdown of specific candidates such as *LPL*, *FABP3*, and *SCD*, are required to determine whether PPAR modulation directly affects viral replication or intestinal injury, and we have outlined these as priorities for future research. Second, the sample size was relatively small, although we applied stringent statistical thresholds and validated key molecules at both protein and metabolite levels to mitigate false-positive risks. Third, our analysis was limited to the jejunum at a single time point; whether similar alterations occur in other intestinal segments or at other infection stages remains unknown. Despite these limitations, our integrative multi-omics approach provides a valuable resource and generates the testable hypothesis that PPAR signaling represents a key correlate of PDCoV-induced metabolic-immune dysregulation. Future mechanistic studies using PPAR-targeted perturbation in susceptible cell lines (e.g. IPEC-J2) are warranted to establish causality.

## Conclusion

Through integrated multi-omics analysis, our findings indicate that PDCoV infection in neonatal piglet jejunum is associated with concurrent activation of antiviral immunity and disruption of the PPAR-regulated metabolic network. This metabolic reprogramming is characterized by widespread membrane phospholipid degradation and alterations in the AA-prostaglandin inflammatory axis, which correlate with intestinal pathology. These parallels with COVID-19 pathophysiology raise the possibility of shared pathogenic features across coronaviruses.

## Material and methods

### Virus and animal ethics

All experiments involving live PDCoV in the present study were conducted in a Biosafety Level 2 (BSL-2) laboratory. The PDCoV strain used (PDCoV/USA/Nebraska145/2015, GenBank: KX022605.1) was isolated from a pig fecal swab in the United States in 2015 and was kindly provided by the Institute of Microbiology, Chinese Academy of Sciences. All animal procedures were approved by the animal ethics committee of Guizhou University (EAE-GZU-2023-E090) and were conducted in strict accordance with the American Veterinary Medical Association (AVMA) guidelines to minimize suffering. No formal protocol was registered prior to the study. The experimental design and analysis plan were documented internally prior to data collection. This study was carried out in compliance with the ARRIVE guidelines (https://arriveguidelines.org). The completed ARRIVE checklist is provided as Supplementary File 2.

### Animal grouping and experimental design

Ten 15-day-old piglets (weighing 3.5–4.2 kg; five males and five females) derived from a PDCoV-negative sow and confirmed free of major enteric viruses, were randomly allocated to two groups using a lottery method (drawing numbered tags from a container): a PDCoV-infected group and a mock-infected control group, with five piglets per group (*n* = 5). Piglets in the infected group were individually numbered 1–5 (three females and two males), and those in the control group were numbered 10–14 (three males and two females). All piglets were confirmed to be PDCoV-negative by qRT-PCR prior to inoculation and showed no clinical signs. No a priori sample size calculation was performed; the sample size was based on previous studies of PDCoV infection in piglets, which have consistently shown that five animals per group are sufficient to observe significant differences in the primary outcomes (intestinal pathology and viral load).

The investigator responsible for group allocation was not involved in animal care, sample collection, or outcome assessment. Data analysis was performed blinded to group allocation. Due to the visible clinical differences (e.g. diarrhea) between infected and control animals, blinding was not possible during the conduct of the experiment or clinical monitoring.

The primary outcome measures were (1) histopathological changes in the jejunum, including villus structure and ultrastructural damage assessed by transmission electron microscopy; and (2) multi-omics profiles (transcriptomic, proteomic, and metabolomic) of jejunal tissues. Secondary outcome measures included clinical signs (diarrhea score) and viral load in jejunal tissues determined by qPCR. The sample size (*n* = 5per group) was based on the primary outcome measures of histopathological changes and viral load, consistent with previous studies.

### Virus inoculation and clinical monitoring

Following a 3-day acclimation period, piglets in the infection group were orally inoculated with 5 mL of virus solution (10^−6.8^ 50% tissue culture infective dose (TCID_50_)/mL), while control piglets received an equal volume of sterile saline. Clinical signs were monitored daily, with fecal consistency scored on a scale from 0 (normal) to 3 (liquid), and rectal swabs were collected for viral RNA qualitative via PDCoV-specific qRT-PCR. Humane endpoints were defined a priori as (1) weight loss > 20% of initial body weight, (2) inability to stand or feed for >12 hours, or (3) moribund state. Any animal meeting these endpoints would be euthanized immediately. No animals reached these endpoints prior to the scheduled euthanasia at 3 dpi. All piglets that met the criteria for successful infection developed moderate to severe diarrhea (scores 2 to 3) and exhibited significant viral shedding by 1 day post-inoculation (dpi), indicating successful establishment of PDCoV infection. At 3 dpi, all piglets were humanely euthanized using an intravenous injection of pentobarbital sodium at a dosage of 50 mg/kg of body weight, in accordance with the guidelines of the AVMA.

### Sample collection and selection for multi-omics analysis

During necropsy, multiple intestinal segments (duodenum, jejunum, ileum, and colon) were collected. Gross and histopathological examination confirmed that the jejunum was the most severely affected segment and was therefore selected for multi-omics analysis. A segment from the middle jejunum was aseptically collected from each animal, immediately placed on ice, opened longitudinally, rinsed gently with ice-cold PBS, and dissected into aliquots.

Specifically, one piglet (No. 1) was excluded from multi-omics analysis because it exhibited low viral load at the experimental endpoint, with qPCR signals that were inconsistent and poorly reproducible across technical replicates, despite showing mild diarrhea, and therefore did not meet the criteria for successful infection. Two control piglets (Nos. 13 and 14) were not included in the multi-omics analysis because three control piglets (Nos. 10–12) were randomly preselected for analysis, based on the uniform health status and minimal within-group variability expected in the control group. No other animals or data points were excluded from the study. These inclusion and exclusion criteria were established a priori. Multi-omics analyses were performed on jejunal samples from infected piglets Nos. 2–5 (*n* = 4) and control piglets Nos. 10–12 (*n* = 3).

### Housing and biosecurity conditions

The experiment was conducted in a BSL-3 laboratory. Prior to the trial, pens and all contact surfaces were thoroughly cleaned, disinfected, and fumigated. Throughout the study, the ambient temperature was maintained at 26–28°C with adequate ventilation. To prevent cross-contamination, piglets in the infected and control groups were housed in separate rooms under identical conditions. Milk replacer was provided five times daily, and feeding equipment and pens were disinfected after each feeding. Pens were cleaned twice daily with alternating disinfectants. Piglets were examined daily for feeding behavior, general health, and clinical signs such as diarrhea, and all observations were recorded.

### TaqMan real-time qualitative PCR (qPCR)

Total RNA was extracted using a TaKaRa MiniBEST Viral RNA/DNA Extraction Kit (Takara, Japan), and cDNA was prepared using the StarScript Pro All-in-one RT Mix with gDNA Remover (GenStar), both in accordance with the manufacturer’s instructions. TaqMan RT-qPCR was performed using 2×RealStar Fast Probe Mix (GenStar) for the qualitative detection of PDCoV in fecal samples from control and infected groups. Positive and negative controls were included in each assay. The probe specifically targeted the N gene sequence of PDCoV. The qPCR reaction was conducted in a 20 μL reaction volume, consisting of 0.4 μL cDNA, 0.5 μL each of forward and reverse primers, 0.5 μL probe, 10 μL 2×RealStar Fast Probe Mix, 0.4 μL High/Low ROX Reference Dye, and sterile water to make up the final volume to 20 μL. A two-step thermal cycling program was employed, with the following conditions: pre-denaturation at 95°C for 2 min, followed by 45 cycles of denaturation at 95°C for 15 s and annealing-extension at 60°C for 20 s. The primer and probe used in this study were synthesized by Sangon Biotech (Shanghai, China) and are listed in Supplemental Table S3.

### Hematoxylin and eosin (HE) staining

Jejunal tissues from control and PDCoV-infected piglets were fixed in 4% paraformaldehyde, embedded in paraffin, and sectioned. Following dewaxing and rehydration, sections were stained with hematoxylin and eosin (HE) using a standard protocol. After dehydration through a graded ethanol series and clearing in xylene, sections were mounted with neutral balsam. HE staining was performed by Wuhan Servicebio Biotechnology Co., Ltd. (Wuhan, China). Morphological examination was conducted under a Nikon 80i upright light microscope (Nikon, Japan), with nuclei stained blue and cytoplasm red. Images were captured using a NIS-Elements BR5.01 camera system.

For quantitative morphometric analysis, at least 10 well-oriented villi and their associated crypts per animal were measured from H&E-stained jejunal sections using ImageJ software (National Institutes of Health, Bethesda, MD, USA). Villus height was defined as the distance from the villus tip to the villus-crypt junction, and crypt depth as the distance from the villus-crypt junction to the crypt base. The villus height-to-crypt depth ratio (V/C) was then calculated accordingly. All measurements were performed in a blinded manner by two independent observers.

### Transmission electron microscopy (TEM) analysis

Jejunal samples from control and PDCoV-infected piglets were fixed with 2.5% glutaraldehyde and post-fixed with 1% osmium tetroxide. Following dehydration through a graded ethanol series, samples were embedded in Epon-812 resin. Semi-thin sections were first examined by light microscopy to identify regions of intestinal epithelium. Subsequently, ultrathin sections (60–90 nm) were collected on copper grids, stained with uranyl acetate and lead citrate, and observed under a JEM-1400FLASH transmission electron microscope (JEOL, Japan). All TEM procedures, from fixation to imaging, were conducted by Chengdu Liliai Biotechnology Co., Ltd. (Chengdu, China).

### Transcriptome sequencing and analysis

Total RNA was extracted from jejunum tissues of three control and four PDCoV-infected piglets using Trizol reagent (Invitrogen, USA). RNA purity and integrity were confirmed with a NanoPhotometer® spectrophotometer (IMPLEN, CA, USA) and an RNA Nano 6000 Assay Kit on a Bioanalyzer 2100 system (Agilent Technologies, CA, USA), respectively. Sequencing libraries were then prepared using the NEBNext® Ultra™ mRNA Library Prep Kit for Illumina® (NEB, USA). After quality assessment, the libraries were sequenced on an Illumina HiSeq™ 4000/Nova platform to generate 150 bp paired-end reads.

Raw sequencing data were filtered with fastp (v0.20.0) to obtain high-quality clean reads. These reads were aligned to the reference genome using HISAT2 (v2.2.1). The resulting alignments were sorted and converted to BAM format using samtools (v1.15.1). Gene expression was quantified with featureCounts (v2.0.1), and the fragments per kilobase of transcript per million mapped reads (FPKM) expression matrix was generated using StringTie (v2.1.6). DEGs were identified with DESeq2 (v1.32.0) using the thresholds of |log_2_(fold change)| >1 and *p*-value < 0.05. Subsequently, Gene Ontology (GO) and Kyoto Encyclopedia of Genes and Genomes (KEGG) enrichment analyses of the DEGs were performed using the clusterProfiler R package (version 4.0.0). All transcriptome sequencing and bioinformatic analyses were performed by Chongqing Life Origin Technology Co., Ltd. (Chongqing, China).

### DIA-proteomics and analysis

For DIA-based proteomic analysis, the same jejunum tissues as described in Sample collection and selection for multi-omics analysis were processed. Proteins were extracted from tissue samples using lysis buffer, followed by homogenization and ultrasonication. Protein concentration was determined via a bicinchoninic acid (BCA) assay. For each sample, 30 μg of protein was reduced, alkylated, and digested with trypsin. The resulting peptides were desalted and vacuum-dried.

Approximately 200 ng of peptides were separated by nanoflow liquid chromatography on a Vanquish neo UPLC system (Thermo Scientific) using an EASY-Spray™ C18 column (150 μm × 15 cm) with a gradient of 0.1% formic acid in water and 0.1% formic acid in 80% acetonitrile. Mass spectrometric analysis was performed on an Astral mass spectrometer operated in data-independent acquisition (DIA) mode. Full MS scans (380–980 m/z) were acquired at a resolution of 240,000, followed by MS/MS scans (150–2000 m/z) with an isolation window of 2 m/z and normalized collision energy of 25%.

Raw DIA data were processed using DIA-NN (version 1.8.1) for protein identification and quantification. DEPs were identified using the limma R package with thresholds of |log_2_(fold change)| >0.263 and *p*-value < 0.05, and subsequently subjected to functional enrichment analysis based on GO and KEGG databases. All proteomic sequencing and bioinformatics analyses were conducted by Chongqing Life Origin Technology Co., Ltd. (Chongqing, China).

### Untargeted metabolomics and analysis

Untargeted metabolomic profiling was performed on the same tissue set (Sample collection and selection for multi-omics analysis) by LC-MS/MS. Metabolites were extracted from approximately 20 mg of homogenized tissue using a 70% methanol aqueous solution containing internal standards. After vortexing, incubation on ice, and centrifugation, the supernatant was collected for LC-MS/MS analysis.

Chromatographic separation was achieved on a Waters ACQUITY Premier HSS T3 column (1.8 µm, 2.1 mm × 100 mm) with a mobile phase gradient consisting of 0.1% formic acid in water and acetonitrile. Mass spectrometric analysis was conducted on a Q Exactive HF-X instrument equipped with an electrospray ionization source, operating in both positive and negative ionization modes. Full-scan MS and data-dependent MS/MS spectra were acquired.

Raw data were processed using XCMS for peak detection, alignment, and retention time correction. Missing values were imputed, and features with a coefficient of variation (*CV*) >0.3 in quality control (QC) samples were removed. Putative metabolite annotation was conducted using a tiered strategy, involving spectral matching against in-house, public (e.g. HMDB, MassBank), and predictive metabolite databases. Differential analysis was carried out by applying a variable importance in projection (VIP) score > 1.0 from Orthogonal Partial Least Squares-Discriminant analysis (OPLS-DA) combined with a *p*-value < 0.05 (from Student’s *t*-test). Biological interpretation of the DEMs was conducted through pathway enrichment analysis based on the KEGG database. All experimental and analytical procedures were performed by Chongqing Life Origin Technology Co., Ltd.

### Integrative multi-omics bioinformatics analysis

An integrated analysis was performed to identify consensus and interacting molecular events across the transcriptome, proteome, and metabolome.

#### Transcriptome-proteome integration

The overlap between DEGs and DEPs was visualized using a Venn diagram. The shared set (denoted as the Share set) was subjected to GO and KEGG enrichment analysis. The global concordance between mRNA and protein abundance was assessed by Pearson correlation. Furthermore, a nine-quadrant scatter plot based on log_2_(fold change) values was employed to identify genes with congruent directional changes across both omics layers.

#### Omics-metabolome integration

Pairwise Pearson correlations were calculated between the expression levels of significant DEGs or DEPs and the abundance of differentially expressed metabolites (DEMs). For the global correlation network, correlations with |*r*| >0.95 and *p* < 0.05 were considered significant. For the focused subnetwork analysis of the PPAR signaling hub and its linked metabolites, a relaxed threshold of |*r*| >0.2 and *p* < 0.05 was applied to better capture potential biologically relevant interactions.

#### Tri-omics integration

KEGG pathways commonly enriched across the transcriptomic, proteomic, and metabolomic datasets were identified by overlapping enrichment results from the three omics layers.

### qRT-PCR validation analysis

To detect the gene expression levels of *FABP3*, *LPL*, *SCD*, *SLC27A2*, *IFIT2*, *OASL*, *MX1* and *RSAD2*, total RNA was isolated from three control and four infected jejunum samples using TRIzol reagent (Invitrogen), according to the manufacturer’s instructions. cDNA was synthesized from 1 µg of RNA using the PrimeScript^™^ RT reagent Kit with gDNA Eraser (Takara, Japan). qRT-PCR was performed with PerfectStart® Green qPCR SuperMix (TransGen) following the manufacturer’s protocols. The qPCR reaction was conducted in a 10 μL reaction volume, consisting of 1 μL cDNA, 0.2 μL each of forward and reverse primers, 5 μL 2×PerfectStart® Green qPCR SuperMix and 3.6 μL ddH_2_O. A two-step thermal cycling program was employed, with the following conditions: pre-denaturation at 94°C for 30 s, followed by 40 cycles of denaturation at 94°C for 5 s and annealing-extension at 60°C for 30 s. Each sample was run in triplicate for analysis. At the end of the PCR cycles, melting curve analysis was performed to validate the specific generation of the expected PCR product. *GAPDH* gene was selected as reference gene, and the designed primer sequences were synthesized by Tsingke Biotechnology Co., Ltd. (Chongqing, China) and are shown in Table S4. The expression levels of mRNAs were normalized to *GAPDH* and calculated using the 2^−ΔΔCt^ method.

### Western blotting analysis

To detect the protein expression levels of MX1, RSAD2 and FABP3, different jejunum samples were lysed in RIPA buffer (Sigma-Aldrich) supplemented with protease inhibitors to extract total protein. Protein samples were separated by SDS-PAGE and transferred onto PVDF membranes (Millipore). Membranes were blocked with 2.5% nonfat milk and incubated overnight at 4°C with primary antibodies. After washing, membranes were incubated with corresponding secondary antibodies. Protein bands were visualized using an enhanced chemiluminescence (ECL) detection system (Thermo Fisher Scientific). The pre-stained protein ladder (Thermo Fisher Scientific, 26,616) was used as a molecular weight standard. For immunoblotting, the following antibodies were used: MX1 monoclonal antibody (1:1000, 13,750–1-AP, Proteintech), RSAD2 monoclonal antibody (1:4000, 68,604–1-Ig, Proteintech), and FABP3 antibody (1:2000, ARP47483_P050, Avivasysbio). β-actin monoclonal antibody (1:5000, 66,009–1-Ig, Proteintech) was used to stain β-actin as a loading control.

### Enzyme-linked immunosorbent assay (ELISA)

The contents of betaine, creatine, arachidonic acid (AA), and prostaglandin E2 (PGE2) in jejunal samples were quantified and verified by ELISA using the Betaine Content Detection Kit (BC3135, Solarbio), Amplex Red Creatine Assay Kit (S0283S, Beyotime), Elabscience® AA ELISA Kit (E-EL-0051, Elabscience), and Elabscience® PGE2 ELISA Kit (E-EL-0034, Elabscience), respectively, strictly following the manufacturer’s instructions of the corresponding kits. Jejunal samples were prepared per kit protocols to preserve metabolite integrity. Reagents were equilibrated to room temperature in advance, and all assay steps (sample loading, incubation, washing, substrate addition, and stop solution addition) were performed precisely as instructed to minimize errors.

Post-reaction, the absorbance (OD value) of each well was measured at the kit-specified wavelength using a microplate reader. Standard curves were generated from kit-provided standards, and metabolite concentrations were calculated accordingly. All assays were run in triplicate to ensure result reliability.

### Statistical analyses

Statistical analyses were performed using GraphPad Prism 10 and SPSS 26. Data are presented as mean ± SD from at least three independent experiments. Data normality was assessed using the *Shapiro–Wilk* test. For comparisons between two groups, unpaired two-tailed Student’s *t*-test was used for normally distributed data, and the *Mann–Whitney U* test was used for non-normally distributed data. For multiple comparisons, one-way ANOVA with Tukey’s post hoc test was used. Pearson correlation was used for linear correlations. Statistical significance was set at *p*-value < 0.05.

## Supplementary Material

Figures.zip

clean260159896_R1_Revised_Supporting_information.docx

## Data Availability

The datasets generated and analyzed during the current study have been deposited in the following public repositories: RNA-seq data have been deposited in the NCBI BioProject database under accession number PRJNA1447169 and are accessible at: https://www.ncbi.nlm.nih.gov/bioproject/PRJNA1447169. Proteomics raw data have been deposited to the ProteomeXchange Consortium via the PRIDE partner repository under dataset identifier PXD076809 and are accessible at: https://www.ebi.ac.uk/pride/archive/projects/PXD076809. Metabolomics data have been deposited to MetaboLights under accession number MTBLS14236 and are accessible at: https://www.ebi.ac.uk/metabolights/MTBLS14236. The raw numerical data supporting the charts and graphs presented in this study (including qPCR Ct values and expression levels, Western blot densitometry, ELISA readings, and statistical analysis outputs) have been deposited in the Science Data Bank (ScienceDB) and are publicly available. DOI: 10.57760/sciencedb.35310. Dataset URL: https://doi.org/10.57760/sciencedb.35310 [64].
