# Supplementary material for: Integrated multi-omics analysis identifies PPAR signaling network associations with metabolic-immune dysregulation and intestinal injury in PDCoV-infected piglets
Source: Virulence. 2026 Sep 27;17(1):2739013. doi: 10.1080/21505594.2026.2739013 (PMC13625717; doi:10.1080/21505594.2026.2739013)
Supplement: Supplementary file 1 [file KVIR_A_2739013_SM5457.docx]

**Integrated Multi-omics Analysis Identifies PPAR Signaling Network Associations with Metabolic-Immune Dysregulation and Intestinal Injury in PDCoV-Infected Piglets**

Xingchen Guo^1#^, Chao A^2, 3#^, Yijie Ding^1^, Yudan Zhang^1^, Haijuan Zhang^1^, Jinyong Wang^2, 3^, Yiyu Zhang^1*^, Tao Wang^2, 3*^

^1^College of Animal Science/Key Laboratory of Animal Genetics, Breeding and Reproduction in the Plateau Mountainous Region, Ministry of Education, Guizhou University, Guiyang 550025, China

^2^Chongqing Academy of Animal Science, Rongchang, Chong Qing 402460, China

^3^National Center of Technology Innovation for Pigs, Rongchang, Chong Qing 402460, China

**Correspondence:**

Yi-yu Zhang, College of Animal Science/Key Laboratory of Animal Genetics, Breeding and Reproduction in the Plateau Mountainous Region, Ministry of Education, Guizhou University, Guiyang 550025, China.

Email: [zyy8yyc@163.com](mailto:zyy8yyc@163.com)

Tao Wang, National Center of Technology Innovation for Pigs, Chongqing Academy of Animal Science, Rongchang, Chong Qing 402460, China.

Email: cqwt101@163.com

1. **Tables and Figures**

**Table S1 Sequences of primers and probe for PDCoV TaqMan RT-qPCR assay.**

| Name | Sequence  (5' to 3') | Amplicon Size  (bp) | Genomic Position  (nt) | Type |
| --- | --- | --- | --- | --- |
| PDCoV-F | GACACTGAGAAGACGGGTATG | 146 | 24625-24645 | Primer |
| PDCoV-R | GTGATTGAGTARGAGAAGGTAAGG | 146 | 24770-24747 | Primer |
| PDCoV-P | FAM-ATGTGCCTGGTGTTCAGGAAATGC-BHQ1 | 146 | 24677-24700 | Probe |

**Footnote:** Nucleotide positions refer to the PDCoV reference genome (GenBank accession no. KX022605.1). The probe was modified with 5'-FAM and 3'-BHQ-1. The “R” in the primer sequence denotes a degenerate base (A/G).

**Table S2 RNA-seq reads filtering summary.**

| Group  name | Sample  name | Total raw  reads (Gb) | Total clean  reads (Gb) | Total clean  bases (Gb) | Clean reads  Q20 (%) | Clean reads  Q30 (%) | Clean reads  ratio (%) |
| --- | --- | --- | --- | --- | --- | --- | --- |
| Control | JEJ_10 | 96.21 | 94.05 | 14.02 | 98.72 | 95.44 | 97.77 |
|  | JEJ_11 | 88.48 | 86.50 | 12.90 | 98.70 | 95.37 | 97.76 |
|  | JEJ_12 | 91.33 | 89.28 | 13.31 | 98.71 | 95.38 | 97.76 |
| PDCoV-infected | JEJ_2 | 85.05 | 83.46 | 12.46 | 98.88 | 95.72 | 98.13 |
|  | JEJ_3 | 89.03 | 87.40 | 13.04 | 98.89 | 95.73 | 98.16 |
|  | JEJ_4 | 85.09 | 83.68 | 12.48 | 98.97 | 95.89 | 98.34 |
|  | JEJ_5 | 88.74 | 87.17 | 13.00 | 98.92 | 95.73 | 98.23 |

**Table S3 RNA-seq mapping statistics against reference genome.**

| Group name | Sample name | Total clean reads (%) | Total mapping genome ratio (%) | Uniquely mapping genome ratio (%) |
| --- | --- | --- | --- | --- |
| Control | JEJ_10 | 94.06 | 95.10 | 89.00 |
|  | JEJ_11 | 86.50 | 95.08 | 89.14 |
|  | JEJ_12 | 89.28 | 95.10 | 88.89 |
| PDCoV-infected | JEJ_2 | 83.46 | 95.54 | 90.33 |
|  | JEJ_3 | 87.40 | 95.50 | 90.98 |
|  | JEJ_4 | 83.68 | 94.98 | 89.53 |
|  | JEJ_5 | 87.17 | 95.17 | 89.58 |

**Table S4 Sequences of primers and probe for qRT-PCR validation analysis.**

| Name | Sequence (5' to 3') |
| --- | --- |
| FABP3-F | CACAAAGCACCTTCAAGAGCAC |
| FABP3-R | AAGTTTGCCTCCATCCAGTGTC |
| IFIT2-F | TCGCCTGAATCCTGACAACC |
| IFIT2-R | CTCTGCCGTCTTCTGCGTTT |
| LPL-F | GCCCTACAAAGTCTTCCATTACC |
| LPL-R | GAATCACTGACCCATTTGAGTTTC |
| MX1-F | CTCATAGACCTTCCTGGCATCA |
| MX1-R | TCTTGTCCTCAGTGCCTTTGTC |
| OASL-F | GCTAGACACCTTCGTGGCTAAAT |
| OASL-R | CTTCTTCACGGTCCTCACAGC |
| RSAD2-F | GCTTGGTGCCCGAGTCTAAC |
| RSAD2-R | TCCGCCCGTTTCTACAGTTC |
| SCD-F | TGGGCGTTTGCCTACTATCTG |
| SCD-R | TCTTTGACGGCTGGGTGTTT |
| SLC27A2-F | TGGCGGTCTTCATGGGTAAT |
| SLC27A2-R | GCTTGTAGATCTGGCGAGGC |
| GAPDH-F | GGTGAAGGTCGGAGTGAACG |
| GAPDH-R | TGGGTGGAATCATACTGGAACA |

**Figure S1**

**Figure S1. Identification of proteins in jejunal tissues by DIA‑proteomics.**
